# Use of *Chlorella vulgaris* Lipidic Extracts in the Development of Healthier Pastry Products with Reduced Fat Contents

**DOI:** 10.3390/foods13233913

**Published:** 2024-12-04

**Authors:** Tatiana Pereira, Sónia Barroso, Paula Teixeira, M. Rosário Domingues, Tatiana Maurício, Susana Mendes, Filipa R. Pinto, Catarina D. Freire, Gabriela Matos, Jorge A. Saraiva, Maria M. Gil

**Affiliations:** 1 MARE—Marine and Environmental Sciences Centre/ARNET—Aquatic Research Network, ESTM, Polytechnic of Leiria, Cetemares, 2520-620 Peniche, Portugal; tatiana.m.pereira@ipleiria.pt (T.P.); sonia.barroso@ipleiria.pt (S.B.); susana.mendes@ipleiria.pt (S.M.); filipa.pinto@ipleiria.pt (F.R.P.); catarina.d.freire@ipleiria.pt (C.D.F.); 2 Universidade Católica Portuguesa, CBQF—Centro de Biotecnologia e Química Fina—Laboratório Associado, Escola Superior de Biotecnologia, Rua Diogo Botelho 1327, 4169-005 Porto, Portugal; pcteixeira@ucp.pt; 3 CESAM, Departamento de Química, Universidade de Aveiro, Campus Universitário de Santiago, 3810-193 Aveiro, Portugal; mrd@ua.pt (M.R.D.); tatianascm97@ua.pt (T.M.); 4 LAQV-REQUIMTE, Department of Chemistry, University of Aveiro, 3810-193 Aveiro, Portugal; gabrielamatos@ua.pt; 5 CoLAB +ATLANTIC, Museu das Comunicações, Rua do Instituto Industrial 16, 1200-225 Lisboa, Portugal

**Keywords:** brioche, rice cake, *Chlorella vulgaris*, lipid extracts, fat reduction

## Abstract

Pastry products constitute a significant segment of the food market. However, the high amount of fat used in their production poses a challenge when competing for the attention of modern consumers, who are more conscious of the health problems associated with the consumption of high-fat products. With this in mind, the main objective of this study is the reduction of the total fat and saturated fat contents of two bakery products, brioche-type bread and rice cake, by partial substitution of the main fat source with *Chlorella vulgaris* lipid extracts obtained through non-thermal high-pressure extraction (HPE). A reduction of 3% in the fat content of the brioche and a reduction of 11.4% in the total fat content of the rice cake were observed when the microalgae extracts were used to replace 10% of the margarine used in the brioche and 20% of the sunflower oil used in the rice cake. This substitution resulted in fat-reduced bakery products with similar physicochemical and nutritional properties to the full-fat controls. A triangle test demonstrated that no differences were perceived for the fat-reduced brioche, while in the rice cake, only slightly perceptible differences were detected. Moreover, brioche and rice cake containing the extract presented values of 1.22 ± 0.27 and 1.29 ± 0.39 mg GAE/g of total phenolic compounds, respectively. DPPH and FRAP activities were also quantified in 0.95 ± 0.38 and 1.83 ± 0.27 µmol AAE/g for brioche with extract and 1.10 ± 0.61 and 1.39 ± 0.39 µmol AAE/g for the rice cake with extract, respectively. The products were microbially stable for at least four days at room temperature. This study demonstrates the potential of using HPE microalgal lipid extracts as fat substitutes in bakery products.

## 1. Introduction

Nowadays, there is increased interest in the reduction of fats present in foods due to the number of health diseases that can arise when following high-fat diets, especially high saturated fatty acids (SFA) consumption. High SFA has been associated with an increased risk of developing cancer and cardiovascular and coronary heart diseases [1,2]. On the other hand, increased consumption of unsaturated fatty acids (UFA) has been shown to decrease the risks of developing these diseases as well as lower mortality [1,2].

The reduction of SFA consumption can be achieved with the use of alternative fat sources richer in UFA that could contribute to reducing the SFA contents and increase the concentration of monounsaturated fatty acids (MUFA) and polyunsaturated fatty acids (PUFA) of foods [3,4]. Microalgae are a rich source of fatty acids, especially PUFA that are essential for a healthy diet [5,6]. Therefore, microalgae lipid extracts could be used to enrich food products or even to substitute unhealthy fat used in some foods.

Nevertheless, the application of microalgae lipid extracts presents some obstacles, such as the relatively low extraction yields and the non-food grade nature of the most effective extraction solvents, which makes them difficult to use at an industrial level [7]. In an effort to overcome these issues, some studies coupled food-grade solvents with novel extraction methods such as ultrasounds, non-thermal high-pressure extraction (HPE), and electric fields [7,8,9,10]. High-pressure processing (HPP) is being increasingly used in food industries as a non-thermal pasteurization method to inactivate microorganisms and increase the shelf life of foods [11]. In addition, this technology has also been studied for a wide range of other applications, such as the recovery of bioactive compounds through HPE, to improve the bioaccessibility/availability of nutrients, to preserve lipids, and to reduce the salt content in food, and reduce allergenicity and the formation of contaminants during processing [11]. HPE can yield food-grade microalgae lipid extracts efficiently and sustainably, preserving the compounds with functional importance. Large-scale application of HPE faces some challenges, including the initial high capital, operational costs, and competition with conventional methods already widely used. However, the growing demand for clean-label products and the ability to obtain high-value compounds can surpass those drawbacks, and HPE can have a positive economic value.

One of the major food industries where fat reduction is necessary is the bakery/pastry industry, which encompasses a large number of high-calorie products with low nutritional value. As previously mentioned, increased consumer awareness of the health problems associated with the consumption of high-fat products can lead to the rejection of these products if these issues are not addressed. In bakery products, the high fat content present, especially SFA, is mainly due to the large amount of butter, margarine, lard, or oil used in their production [12,13]. Thus, reducing the amount of fat present is of utmost importance. However, fat reduction can have an impact on product properties, as fat plays an important role in product characteristics, specifically texture, by improving the lubrication of gluten particles and creating smoother and more uniform shapes upon baking, and in the sensory domain, by providing flavor through the aeration of the dough and the lubricating effect on the palate [14,15]. It also contributes to delayed starch gelatinization, increasing the shelf life of the product [14,15].

Several strategies to reduce and/or substitute the fat source have been investigated, such as the use of alternative ingredients with the potential to mimic fat’s effects on the products, like complex carbohydrates (e.g., rice starch, inulin, among others), gums and gels (e.g., xanthan gum, guar gum, pectin, among others), oleo gels (e.g., olive, soybean, and/or canola oils structured with candelilla, bee, and/or sunflower waxes), whole foods (e.g., avocado puree, apple puree, chia seed mucilage, among others), and combinations of ingredients (e.g., polydextrose/guar gum, maltodextrin/xanthan gum, among others) [4,16]. Each influences the bakery products in different ways and, thus, can induce distinct changes in the quality of the final product [16].

The use of lipid-based substitutes allows the production of low-fat products with the least impact on taste [17]. Liquid oils with higher amounts of unsaturated fats could be the perfect substitute; however, they will result in products with noticeable sensory changes [18,19] that could hinder their acceptance by the consumer. Considering this, focus has been directed to the creation of liquid-based oleogels. Their application allows for significant reductions in the fat content of bakery products like croissants [19,20], cookies [20,21], sponge cake [20,22], and muffins [20], among others. Sunflower, olive, and canola oil are often used in these studies.

Microalgae has the potential to be considered as an alternative to the more common oilseed crops due to the fact that it can be produced rapidly and continuously all year round with no need for arable land [23,24]. They are also able to produce high amounts of lipids and polyunsaturated fatty acids, such as LA (18:2), ALA (18:3), EPA (20:5), and DHA (22:6) [23,25]. However, they still present various disadvantages, such as cost of production, energy, and water requirements [24]. Algae have been used in foods as a way to increase the protein, fiber, minerals, and omega-3 fatty acids contents and in the creation of healthier products [5,25,26].

The main objective of this study is to evaluate the potential of using microalgal lipid extracts in the development of healthier bakery products through partial fat replacement in brioche-type bread and rice cake using *Chlorella vulgaris* lipid extracts obtained by non-thermal HPE. It has been seen that the effect of the fat replacement depends on the type of bakery product [3], and thus, two bakery products were selected as models for fat substitution: brioche-type bread and rice cake. Rice cake (also known as Portuguese rice cake, rice muffin, or *bolo de arroz*) was chosen because of its popularity in Portugal and its high fat content, with values around 14.8 g/100 g [13,27].

## 2. Materials and Methods

### 2.1. Microalgae Extracts

*Chlorella vulgaris* (White), used to produce the extracts, was provided by Allmicroalgae (Pataias, Portugal). Microalgae extracts were obtained by non-thermal high-pressure extraction (HPE). For extraction, microalgae powder was suspended at 0.5% (*w*/*v*) in ethanol 96%. The mixture was then placed in low permeability polyamide–polyethylene bags (PA/PE-90, Ideiapack, Viseu, Portugal) and heat-sealed manually, leaving a minimum amount of air inside the bags. HPE was performed in a 55 L capacity industrial-scale high-pressure equipment (model 55, Hiperbaric, Burgos, Spain) at 420 MPa for 9 min, using water as a pressure-transmitting medium and carried out at ambient temperature (~20 °C). Parameters of the pressurization cycle, such as compression rate, were fixed at 250 MPa/min, while complete decompression took about 10 s. After extraction, samples were filtered with standard filter paper (ST61 150 mm, JMGS, Odivelas, Portugal). Then, the collected filtrate solution was concentrated by evaporation using a rotary evaporator (Buchi Rotavapor R-210, Rotoquímica, Portugal) and finally dried in a laboratory dryer oven under controlled temperature (40 °C) to obtain the oily extract that was stored until use at −18 °C.

### 2.2. Preparation of the Bakery Products

#### 2.2.1. Brioche-Type Bread

A recipe for brioche-type bread was chosen as a model for reducing the fat content in pastry products [28]. The quantities of the ingredients used in the production of the control and the reduced fat brioche are presented in Table 1. In the second brioche, the fat was partially replaced by lipidic *C. vulgaris* extract.

In a food processor (Bimby^®^ TM6, Vorwerk, Carnaxide, Portugal), the dry ingredients were mixed with the eggs at low speed (1) for 5 min, followed by 10 min at high speed (2.5). Melted margarine and milk were added, and the dough was mixed using the knead function until the dough loosened from the sides of the bowl (10 min). The dough was leavened on a greased and lightly floured tray for one and a half hours and covered with a cloth. The brioche was baked in a pre-heated oven (180 °C) for 22 min (turning on the overhead heating for the last 3 min) and placed on a wire rack to cool.

For the preparation of the brioche with lipidic extract, 10% of the margarine was substituted with lipidic *C. vulgaris* extract at a concentration of 0.13% in the brioche bread. In this case, the microalgae extract was previously dissolved in the melted margarine and milk mixture.

Three brioches of each formulation (control and brioche with extract) were produced on the same day for the physical–chemical and nutritional analysis.

Analyses of baking loss, pH, a_w_, texture, and moisture were carried out on fresh samples, while the analyses of protein, fat, fatty acid profile, and antioxidant activity were performed on freeze-dried samples.

#### 2.2.2. Rice Cake

A traditional recipe for the rice cake was used [29], and the ingredients can be found in Table 2.

Rice flour was obtained using whole rice pulverized in a food processor (Bimby^®^ TM6) for 2 min (speed 9) and stored until use. Sugar and eggs were mixed in the food processor for 2 min (speed 3), and then milk and melted butter were added and mixed for a further 3 min at the same speed. The butterfly accessory was installed on the food processor, and the flours (wheat and rice) and yeast were mixed with the ingredients for 2 min (speed 2). Lastly, the oil was mixed into the dough for an additional 2 min (speed 2).

For the preparation of the rice cake with lipidic extract, 20% of the sunflower oil was substituted with lipidic *C. vulgaris* extract at a concentration of 0.11%. In this case, the microalgae extract was previously dissolved in the melted margarine and oil.

The dough was placed in metal rings lined with parchment paper and baked at 160 °C for 30 min. Sugar was sprinkled on the cakes after 20 min of cooking.

The baked rice cakes were removed from the metal rings and left to cool before analysis.

### 2.3. Baking Weight Loss, pH, a_w_, and Color

Unless indicated otherwise, all measurements were performed in triplicate.

The baking weight loss was calculated using the formula presented in Equation (1), being m0 the weight before it was placed in the oven and mt the weight 1.5 h after baking [30].

(1) $$\mathrm{Baking}\;\mathrm{Weight}\;\mathrm{Loss}\;\%=\frac{m0-mt}{m0}\times 100$$

The pH values of the dough and baked cakes were obtained using a pH meter (Inolab, WTW, Weilheim, Germany) equipped with a perforation probe, and the a_W_ of the cakes was analyzed with a hygrometer (HP23-AW-A, Rotronic, Bassersdorf, Switzerland) [31]. All measurements were performed in triplicate.

Color analysis was conducted using a Konica Minolta Chroma Meter CR-400 (Tokyo, Japan) at a 2° angle and D65 illuminant. In the brioche, the analysis was performed in triplicate on a portion of the dough and four slices (two from the center and two from the extremities) of the brioche. In the slices, the color was measured at four different locations on the crust and four on the crumb. In the rice cakes, the color was measured on five different cakes from each formulation in one spot on the crust and crumb of each one. L* (black–white, 0–100), a* (green–red, −60–60), and b* (blue–yellow, −60–60) values were used in the calculation of the total color difference (∆E) using Equation (2) [32,33].

(2) $$∆E=\sqrt{\left.\left({∆L}^{2}\right)+\left({∆a}^{2}\right)+({∆b}^{2}\right)}$$

### 2.4. Texture

Texture profile analysis (TPA) of the brioche-type bread samples was evaluated with a texturometer (TA-XTplus, Stable MicroSystems, Surrey, UK) less than 24 h after production. The values were obtained on three center slices (1.5 cm thickness) with four readings from each slice. On the rice cakes, the texture was evaluated on the day of production, and the values were obtained from center slices (1.5 cm thickness) of five rice cakes for each formulation.

The analysis conditions were 30 kg trigger load, 5 g trigger force, 1 mm/s test speed, 5 s waiting time, 10 mm cylinder probe, and 6 mm distance (equivalent to 40%) [32,33]. The software Exponent Connect 6.1.18.0 (Stable Micro Systems, Surrey, UK) was used to extract the values for hardness, resilience, cohesion, and springiness using the “Simplified TPA macro”.

### 2.5. Proximate Chemical Composition

Moisture was analyzed through the difference in the sample weight after an overnight incubation at 105 °C [34]. Ash was calculated after heating the samples at 535 °C for 5 h in a furnace [35].

Crude protein was obtained using the Dumas methodology and a nitrogen factor of 6.25 [36,37].

Total fat was quantified using a slightly modified Folch et al. [38] methodology [28]. A freeze-dried sample (1 g) was mixed with 0.8 mL of water and 5 mL (2:1) of chloroform (99.2%, VWR, Fontenay sous Bois, France)/methanol (99.9%, Carlo Erba, Val-de-Reuil, France) using a vortex (1 min), followed by mixing another 5 mL of chloroform/methanol (2:1). After 5 min, 1.2 mL of 0.8% sodium chloride (Biochem, Cosne-Cours-sur-Loire, France) was added and homogenized for an additional 2 min. The solution was centrifuged for 10 min (6000 rpm, 4 °C), and the lower phase was filtered using hydrophobic cotton and sodium sulfate anhydrous (Honeywell, Seelze, Germany) into a pear-shaped flask. The solvent was evaporated with a rotavapor (Laborota 4000, Heidolph, Scwabach, Germany), followed by oven incubation overnight to ensure total evaporation. Samples were kept in a desiccator until a constant weight was achieved.

### 2.6. Fatty Acid Profile

The fatty acid profile of bakery products was obtained using an acid-catalyzed direct transesterification methodology [39], which involved the dissolution of a fat sample (10 mg) with 2 mL of methanol (Carlo Erba) with 2% of sulfuric acid (Honeywell) at 80 °C in a water bath for 2 h. The solution was then homogenized for 1 min with MiliQ water (1 mL) and 2 mL of n-hexane (Carlo Erba), transferred to a falcon tube, and centrifuged for 5 min (1000 rpm, 4 °C). Then, 1 mL of the upper phase (organic phase) was placed in gas chromatography (GC) vials and analyzed on a GC-FID chromatograph (Finnigan trace GC Ultra, Thermo Scientific, Thermo Electron S.p.A., Milan, Italy) equipped with an autosampler (AS 3000, Thermo Electron Corporation) and a TR-FAME capillary column (Thermo TR-FAME, 60 m × 0.25 mm ID × 0.25 µm film thickness). Helium was used as carrier gas (1.5 mL min^−1^ flow rate), while air and hydrogen (50 mL min^−1^ and 35 mL min^−1^ flow rate, respectively) were supplied to the detector. The temperature used can be found in [28].

The fatty acid profile of the algal extracts obtained by non-thermal high-pressure extraction (HPE) was evaluated by GC-MS analysis of the FAME obtained by alkaline transmethylation [6]. A chromatography-mass spectrometry (GC–MS) (Agilent Technologies 8860 GC System, Santa Clara, CA, USA) was equipped with a DB-FFAP column with the following specifications: 30 m long, 0.32 mm internal diameter, and 0.25 μm film thickness (J&W Scientific, Folsom, CA, USA). The GC equipment was connected to an Agilent 5977B mass selective detector operating with electron impact ionization at 70 eV and a scanning range of *m*/*z* 50–550 (1 s cycle in a full scan mode). The following conditions were used: helium as carrier gas (constant flow 1.4 mL min^−1^), inlet temperature 220 °C, detector temperature 230 °C, and injection volume 2 μL (splitless). The oven temperature was programmed as follows: 58 °C for 2 min, 25 °C min^−1^ to 160 °C, 2 °C min^−1^ to 210 °C, and 30 °C min^−1^ to 225 °C (held for 20 min). The data acquisition software used was GCMS 5977B/Enhanced MassHunter qualitative analysis 10.0.

The FA profile was determined by comparison of retention times with a standard (Supelco 37 component FAME Mix, Sigma-Aldrich Chemie GmbH, Steinheim, Germany). All analyses were performed in triplicate, and the results expressed as % of total FA.

### 2.7. Antioxidant Activity

The antioxidant potential was evaluated by the determination of total phenolic compounds and evaluation of the ability to reduce the DPPH radical and the ferric-reducing ability of the brioches (control and extract brioche) and rice cakes (control and extract rice cakes). First, the extracts were obtained by adding 10 mL of ethanol absolute (99.6%, Aga, Prior Velho, Lisbon, Portugal) to 1 g of lyophilized sample and homogenizing for 5 min using a vortex. The mixture was left to rest overnight at 4 °C in the dark, centrifuged for 15 min (8000× *g*, 4 °C), and the supernatant was transferred to a 15 mL falcon tube. The supernatant was centrifuged a second time (15 min, 8000× g, 4 °C) to ensure that all the biomass was removed.

The total phenolic compounds (TPC) were quantified using an adaptation of the Folin–Ciocalteu methodology [40,41]. Initially, 50 µL of Folin–Ciocalteu reagent (Panreac, Castellar del Vallès, Barcelona, Spain) was added to an aliquot of 10 µL of the extracts diluted with 790 µL of distilled water in Eppendorf tubes and placed in the dark for 2 min at room temperature. Blanks of the reaction were prepared by substituting the Folin–Ciocalteu reagent with distilled water. Then, 150 µL of 20% Na_2_CO_3_ (Scharlau, Sentmenat, Barcelona, Spain) were added, and the samples were placed in the dark for 1 h at room temperature. The samples were transferred to a microplate and the absorbance read at 755 nm. Gallic acid (Merck, Darmstadt, Germany) at different concentrations (1, 0.3, 0.1, 0.03, and 0.01 mg/mL) was used as reference standard, and the results are presented in mg of gallic acid equivalents/g of extract (mg GAE/g) [28].

The ferric-reducing ability of the extracts was evaluated using a modified Benzie and Strain [42] methodology [28]. A working ferric solution of TPTZ was prepared on the day of analysis using acetate buffer, TPTZ, and a ferric solution (10:1:1). Acetate buffer (300 mM, pH 3.6) was prepared with sodium acetate (3.1 g/L, Biochem) and acetic acid (16 mL/L, VWR). The TPTZ solution was prepared by dissolving 10 mM of TPTZ (Sigma, Darmstadt, Germany) in HCl (40 mM, VWR). Lastly, the ferric solution was prepared by dissolving the FeCl_3_·6H_2_O (Scharlau) in distilled water at a concentration of 20 mM. The reaction was conducted in Eppendorf tubes by adding 975 µL of the ferric solution of TPTZ to 25 µL of the extracts, followed by incubation for 4 h in the dark (room temperature). After transfer to a microplate, the absorbance was read at 593 nm. Ascorbic acid (Carlo Erba) was used as reference (1000 µM, 750 µM, 500 µM, 200 µM, and 20 µM), and water was used (instead of the ferric solution of TPTZ) to prepare reaction blanks. Results are expressed as ascorbic acid equivalents µmol/g of extract (µmol AAE/g).

The ability to reduce the DPPH radical was evaluated according to the Brand-Williams et al. [43] method [28]. In Eppendorf tubes, 990 µL of a methanolic solution of DPPH (0.1 mM, Sigma-Aldrich, Darmstadt, Germany) was incubated with 10 µL of the extracts (100 g/L). After 30 min in the dark (room temperature), the samples were transferred to a microplate and the absorbance read at 517 nm. Sample blanks were prepared using methanol instead of the DPPH. Control samples were prepared with ethanol absolute. Ascorbic acid was used as the reference standard (1000 µM, 750 µM, 500 µM, 200 µM, and 20 µM), and the results were expressed as µmol AAE/g.

### 2.8. Microbiological Stability

The microbiological stability of the products was evaluated over a period of 4 days. Each formulation was produced and divided into portions for daily analysis. For each sample, 25 g was homogenized for 2 min with 225 mL of sterile buffered Ringer’s solution (Biokar Diagnostics, Beauvais, France) using a stomacher (Interscience, Saint Nom la Breèteche, France) [28]. Ringer’s solution was used to prepare decimal dilutions of the samples for the enumeration of total viable counts at 30 °C [44], yeast and molds at 25 °C [45] *Bacillus* spp. [46] and *B. cereus* [47].

### 2.9. Sensory Analysis

A triangle test was used to compare the full-fat and the reduced-fat versions of the products. This test was conducted following the ISO 4120 [48] methodology. The test was conducted using a semi-trained panel that was presented with 3 samples identified with a 3-digit random number. The panel was composed of 17 panelists (15 females and 2 males) for brioche and 18 panelists (15 females and 3 males) for rice cake. As for ISO 4120 [48], the samples were randomized in a way to assure that two samples from the same food (A) and one sample from the other (B) were presented to the panelists (in ABB, AAB, ABA, BAA, BBA, and BAB sequences) and they were requested to choose the odd one out. Each panelist was given two triads to assess, and water and crackers were provided to cleanse the palate. After choosing the odd sample, the panelists were asked to indicate the degree of difference as “none”, “slightly”, “moderate”, “much”, or “extreme” differences between the odd one and the duplicates [49]. Significant differences were determined using the minimum number of correct answers found in the binomial table for a significance of 0.05 [48]. A section for comments was included in the question sheet.

### 2.10. Statistical Analysis

All analyses were performed in triplicate and in three different batches of each formulation (with and without fat substitution), with the results presented as mean ± standard deviation (SD). Differences between the physical–chemical, nutritional, and bioactivity results were found using a Student’s *t*-test analysis. All normality of data and homogeneity of variances assumptions inherent to the *t*-test were validated, and whenever these requirements were not met, the Mann–Whitney U test was used instead. Statistical analysis was performed using IBM SPSS Statistics 28 (Copyright IBM Corp. ©1989–2023, Armonk, NY 10504-1722, USA), and the results were considered statistically significant at the 5% significance level.

## 3. Results and Discussion

### 3.1. Effects of the Fat Source’s Partial Substitution on the Physical–Chemical and Nutritional Properties of the Bakery Products

The reduction in the fat content in bakery products was achieved by replacing an amount of margarine and oil used in their production process with *C. vulgaris* extracts obtained using HPE. The relative abundance of the fatty acids present in the extracts used in this study is presented in Table 3.

Initially, theoretical formulations were conducted to evaluate the amount of fat that was possible to replace with the extracts with the intention of reducing the amount of total fat and saturated fat present in the products. Preliminary sensory analyses using various levels of fat substitution helped to define the amount that was possible to replace with minimal sensory impact (preliminary tests done with 4–5 elements of the semi-trained sensory panel).

In brioche-type bread, the main source of fat is the margarine used in production. Sensory analysis of the brioche-type bread showed that it was possible to reduce the SFA by 10% with minimal impact on the product’s sensory properties (preliminary tests done with 4–5 elements of the semi-trained sensory panel). This reduction was obtained through the substitution of 10% of the margarine with *C. vulgaris* lipidic extract at a concentration of 0.13%. On the other hand, for the rice cake, there are two main ingredients that contribute to the fat content, margarine and sunflower oil. Margarine is added in lower amounts and is the main source of saturated fats, while oil is the main contributor to the high fat content of the product. Preliminary sensory analyses showed that the reduction of margarine impacted the final products more negatively than the oil reduction (preliminary tests done with 4–5 elements of the semi-trained sensory panel). Therefore, it was possible to reduce the total fat by 10% without causing negative effects on the product by replacing 19.7% of the sunflower oil with *C. vulgaris* lipidic extract at a concentration of 0.11%.

**Figure 1:**
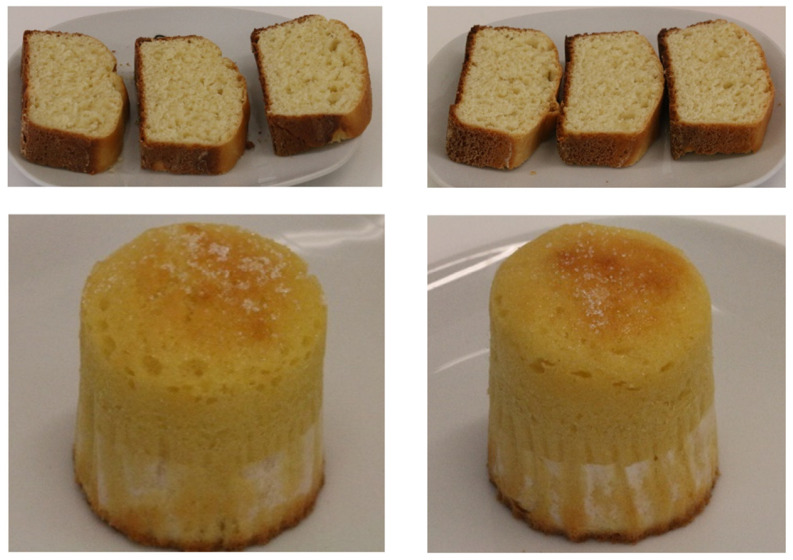
Representation of the control brioche (**upper left**), control rice cake (**lower left**), reduced-fat brioche with lipidic extract (**upper right**), and reduced-fat rice cake with lipidic extract (**lower right**).

Figure 1 shows a representation of the brioche (control and reduced-fat brioche with extract) and the rice cake (control and reduced-fat rice cake with extract), and the results of the physical–chemical and nutritional proximate analysis can be found in Table 4.

The partial substitution of margarine with *C. vulgaris* lipidic extract did not seem to have a major impact on the brioche’s physical–chemical characteristics. Both the control and the reduced-fat brioches presented similar average values for weight loss, pH, resilience, cohesion, springiness, color, protein, and energy values. However, statistically significant differences were verified on the a_W_, hardness, moisture, ash, total fat, and carbohydrates (*p*-value < 0.05), with the reduced-fat brioche presenting higher a_w_, hardness, carbohydrates, and energy values and lower moisture, fat, and ash.

In terms of total fat, the replacement of 10% margarine with *C. vulgaris* lipidic extract resulted in a total fat reduction of 3% in the reduced-fat brioche vs. the control. Therefore, there was a reduction in the total FA, with a decrease of 5.4% of the SFA and 3.7% of the PUFA and an increase of 0.4% of the MUFA in the reduced-fat brioche with lipidic extract. The reduced-fat brioche presented a reduction of 1.2% of the SFA along with an increase of 1.3% of MUFA when compared to the control brioche. The contents of PUFA were maintained. The increase in MUFA may come from the contribution of algae extracts that have an increased content of MUFA (14.1%) with the contribution of the oleic acid (e18:1) and (18:1 n-7).

Comparable results were obtained for the control and the reduced-fat rice cake. Nevertheless, statistically significant differences were verified on the higher weight baking loss, a_W_, carbohydrates, hardness, and springiness and the lower moisture, total fat, energy value, resilience, and cohesion of the reduced-fat rice cake (*p*-value < 0.05).

Regarding total fat, the substitution of the oil with the microalgae lipidic extract resulted in a reduction of 11.4% of the total fat. The total fat reduction resulted in a reduction of 4.1% of the SFA, 12.0% of MUFA, and 15.4% of the PUFA in the reduced-fat rice cake when compared to the control. A redistribution of the FA was also observed, with an increase in the percentage of SFA (2.1%) and a decrease in PUFA (2.1%) in the reduced-fat rice cake compared to the control. The reduction in the UFA of the reduced-fat products when compared to the controls, especially in the reduced-fat rice cake, was expected since sunflower oil has high amounts of unsaturated fatty acids, especially PUFA, and thus, any reduction will impact the product’s FA composition. Considering this, the reduction of the sunflower oil had a negative impact, in terms of UFA, on the nutritional quality of the rice cake. This reduction was also verified by Doménech-Asensi et al. [20] when margarine and sunflower oil were replaced by high-oleic sunflower oil and inulin in cookies, sponge cake, croissants, and muffins. The substitution led to a decrease in total fat (12 to 50%) and SFA but also a decrease in the PUFA [20]. The substitution of PUFA-rich sunflower oil with MUFA-rich oleic sunflower oil was the main reason for this decrease [20]. In the present study, higher concentrations of lipid extract may have aided in mitigating this reduction due to the high PUFA content of *C. vulgaris* extracts [7,50]. An alternative would have been to reduce the amount of margarine used in the preparation of the rice cake, but as mentioned above, reducing the margarine had a higher influence on the texture of the product and any reduction made the product sensorily unacceptable. The addition of ingredients with the ability to mitigate the effects of the margarine reduction may also have aided in further reducing SFA. Various studies have mentioned the inability to replace solid fats with liquid oils due to the functionality provided by the SFA and *trans*-fat [4,51]. Fat plays a major role in trapping air cells during the creaming process, contributing to a soft and tender texture [16,52]. Therefore, one of the most frequent effects of its replacement is an increase in texture parameters, especially hardness [12,16]. The structure of liquid fats is being studied to keep the functionality of solid fats while maintaining the nutritional profile of oils [4]. Gutiérrez-Luna et al. [21] structured olive oil using alginate to totally substitute the butter used in cookies. The reformulated cookies presented a decrease of 40% in total fat and 70% in SFA, but also an increase in hardness and a decrease in sensory acceptability [21]. Moreover, structured emulsions in shortbread cookies as butter substitutes reduced the total fat (by 20.5–32%) and SFA (by 40–75%); however, this was also accompanied by an increase in hardness and a decrease in sensory scores [53].

In this study, the partial replacement of fat with the extracts significantly increased the hardness and springiness and decreased the resilience and cohesion of the reduced-fat rice cakes, while in the reduced-fat brioche, only the hardness increased (*p*-value < 0.05). The low concentration of lipidic extract used was not enough to mimic the effect of the fat on the products; nevertheless, the average texture results were not that different between the control and the partial fat substitution products. The possible creation of oleo gels using the lipidic extract could result in better texture results, but this would require a sustainable scale-up to ensure the availability of the extract. The addition of other ingredients such as gums (e.g., xanthan, gum arabic, guar gum), fibers (e.g., β-glucan, inulin), or proteins (e.g., whey) could have also helped to mitigate the textural effect of the reduction [52].

### 3.2. Antioxidant Activity

The antioxidant activity of the brioche and rice cake was evaluated. Different methodologies were used to elucidate the potential antioxidant activity due to the different mechanisms of action that can be involved in the bioactivities. Thus, the total phenolic compounds were quantified, and the FRAP and DPPH scavenging activity were evaluated. The results are presented in Table 5.

No statistical differences were found in the values of DPPH activity quantified for the two brioches (*p*-value > 0.05). As for the total phenolic compounds, higher total phenolic compounds were obtained for the brioche control (1.55 ± 0.35 mg GAE/g) than for the reduced-fat brioche (1.22 ± 0.27 mg GAE/g) (*p*-value < 0.05). On the other hand, higher ferric-reducing activity was obtained for the reduced-fat brioche, reaching values of 1.83 ± 0.27 µmol AAE/g. However, these values were significantly lower than those obtained for the brioche control (*p*-value < 0.05). While a decrease in the TPC and FRAP activity was observed upon partial fat substitution by lipidic extract, an average higher DPPH activity was observed for the reduced-fat brioche, suggesting that a higher addition of lipidic extract may have contributed to an increase in DPPH-reducing capacity.

As for the rice cakes, the partial fat substitution with lipidic extract did not seem to influence the antioxidant activity of the product. For the reduced-fat rice cake, a profile of 1.29 ± 0.39 mg GAE/g for TPC, 1.10 ± 0.61 µmol AAE/g for DPPH inhibition, and 1.39 ± 0.39 µmol AAE/g for FRAP activity was found, which was like that obtained for the control rice cakes (*p*-value < 0.05). Nevertheless, the results suggest that an increase in the lipidic extract concentration may have contributed to increased bioactivity since the average results of the reduced-fat rice cake’s assays were all above the average results of the control.

It is known that the baking process can significantly reduce the antioxidant activity of products [54]; nevertheless, some activity was quantified. Higher concentrations of lipidic extract could have increased this bioactivity since the antioxidant activity of *C. vulgaris* lipid extracts has been previously reported. *Chlorella vulgaris* extracts obtained with ethanol presented a DPPH IC50 of 108.6 mg/mL [55]. Different extraction solvents, methodologies, and times, as well as different extract concentrations used on the antioxidant assays and possible protocol modifications, could hamper an effective comparison between studies. Despite this, it can be confirmed that *C. vulgaris* extracts present DPPH activity. Moreover, growth conditions can also influence the antioxidant activity of the microalgae, as confirmed in a comparison between *C. vulgaris* produced in auto or heterotrophy conditions. It was verified that the autotrophic growth conditions increased the antioxidant activity for *C. vulgaris* lipidic extracts (IC50 of 110.2 ± 8.5 µmol/g Trolox equivalents) when compared with heterotrophic *C. vulgaris* extracts (IC50 of 120.1 ± 3.9 µmol/g Trolox equivalents) [50].

The incorporation of whole *C. vulgaris* in other products resulted in foods with quantifiable antioxidant activity even after heat treatment, as reported in the literature. *Chlorella vulgaris* biomass in crackers increased the TPC by 21% [56]. Moreover, the use of *C*. *vulgaris* Smooth originated 3D-printed snacks with higher antioxidant activity than the control [57]. However, even obtaining higher bioactivities, one of the main drawbacks of the addition of whole biomass in food is the potential taste conferred on the products. The use of extracts can overcome these sensory disadvantages, but there are other barriers, such as extraction yields and the cost associated with their production.

### 3.3. Sensory Analysis

As previously mentioned, fat is an important ingredient in bakery products, and its reduction can impact the characteristics and acceptability of the food. To determinate if there were perceptible differences between the samples, a triangle test was used (Table 6).

**Figure 2:**
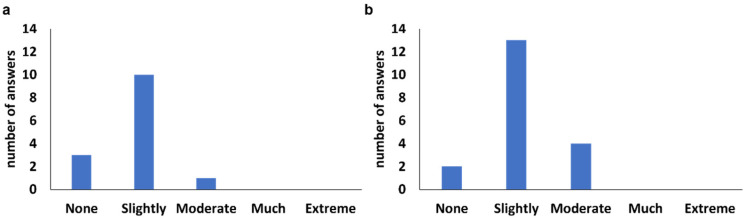
Level of differences (from none to extreme) perceived by the panelists for brioche (**a**) and rice cake (**b**).

According to the binomial table, the minimum number of correct answers to determinate perceptible differences was 17 and 18 for brioche and rice cakes, respectively [48]. As can be seen, in brioche, the number of correct answers was lower than the minimum needed, and thus, it can be concluded that no differences were identified. In the rice cake, there was a higher number than the 18 needed, and it can be said that perceptible differences exist between the control rice cake and the reduced-fat rice cake (*p*-value < 0.05). From the correct responses, the level of differences identified by the panelists was seen (Figure 2).

The majority of the correct assessments indicated that the odd sample presented slight differences (52.6% of the correct answers for brioche and 68.4% for rice cake) from the duplicated samples. The rest of the answers for the brioche denoted none (15.8%) or moderate (5.3%) differences, while for the rice cake, there were 10.5% none and 21.1% moderate differences.

In the reduced-fat versions, the panelists more frequently indicated slight differences in taste, followed by smell and texture. In the reduced-fat rice cake, the taste appeared sweeter than the control with a denser texture. For the brioche, the differences in taste were not as consistent as with the rice cake, with panelists indicating a sweeter taste in both control and reduced-fat versions. A slightly denser texture was also felt for reduced-fat brioche. As mentioned above, both products presented statically significant differences in texture and carbohydrate content that were confirmed in the sensory analysis. These differences were more pronounced in the rice cake. As mentioned above, the use of other strategies, such as oleogels or the addition of other ingredients, could have aided in the replacement of the fat by mitigating the changes in the sensory characteristics of the products [3,52].

### 3.4. Microbiological Stability

The microbial stability of both model products was evaluated during 4 days of storage at room temperature. Bakery products are traditionally consumed as fresh products, thus presenting a short shelf life at room temperature of around 3–5 days [58,59]. For bakery products with low to medium moisture content, shelf life is mainly constrained by physical and chemical spoilage. However, in products where a_w_ > 0.85, microbial spoilage caused by bacteria, yeasts, and molds becomes a significant concern (reviewed by Smith et al. [60]).

The microbial stability was evaluated by monitoring total microbial counts, molds, yeasts, *Bacillus* spp., and *B. cereus* (Table 7). The results indicated that the products maintained acceptable microbial levels during the monitoring period, demonstrating a shelf life of at least 4 days.

## 4. Conclusions

*Chlorella vulgaris* lipidic extracts obtained by HPE were evaluated as a partial replacement of the main fat source in brioche and rice cake. This substitution was successful in the reduction of total fat in both products with a small percentage of lipid extract addition (0.13 and 0.11% in brioche and rice cake, respectively) without affecting the sensory characteristics of the products. Nevertheless, the reduction in total fat was accompanied by a reduction in SFA, but also a reduction in the beneficial MUFA and PUFA. Further studies should focus on the scale-up of the extraction of food-grade lipid extracts to enable a higher percentage of extracts to be added to the products, thus allowing a decrease in SFA and, eventually, an increase in PUFA. The triangle test demonstrated that no differences were found for the reduced-fat brioche. On the other hand, slight differences were perceived in the reduced-fat rice cake.

## Figures and Tables

**Table 1:** Recipes of the control brioche and reduced fat (RF) brioche with lipidic *C. vulgaris* extract.

Ingredients	Control Brioche (g)	Reduced Fat Brioche (g)
Wheat flour	180	180
Baker’s yeast	3	3
Salt	0.25	0.25
Sugar	20	20
Half-skimmed Milk	30	30
Margarine	50	45
Whole egg	58	58
*C. vulgaris* White extract	--	0.44

**Table 2:** Recipes of the control rice cake and reduced-fat (RF) rice cake with lipidic extract.

Ingredients	Control Rice Cake (g)	RF Rice Cake (g)
Rice flour	50	50
Wheat flour	75	75
Dry yeast	7.5	7.5
Sugar	75	75
Half-skimmed Milk	75	75
Margarine	15	15
Whole egg	105	105
Sunflower oil	30	24.1
*C. vulgaris* White extract	--	0.48

**Table 3:** Relative abundance of fatty acids present in the microalgal extract. Data are presented as mean ± SD (*n* = 3).

Fatty Acids	Relative Abundance (% of Extract)
C14:0	0.6 ± 0.1
C15:0	0.2 ± 0.0
C16:0	54.8 ± 3.8
C16:1 (n-7)	1.1 ± 0.3
C15:0 (iso)	4.0 ± 0.3
C16:2 (n-6)	1.5 ± 0.5
C17:0	0.3 ± 0.1
C18:0	22.8 ± 1.5
C18:1 (n-9)	7.6 ± 1.8
C18:1 (n-7)	6.5 ± 2.8
C20:0	0.3 ± 0.0
Σ SFA	83.2 ± 6.6
Σ MUFA	15.3 ± 6.0
Σ PUFA	1.5 ± 0.7

Abbreviations: Σ SFA, the sum of saturated fatty acids; Σ MUFA, the sum of monounsaturated fatty acids; Σ PUFA, the sum of polyunsaturated fatty acids.

**Table 4:** Physical–chemical and nutritional proximate analysis of the brioche and rice cake controls (full fat) and the reduced-fat brioche (RF Brioche) and rice cake (RF Rice cake) with partial replacement of fat by *C. vulgaris* lipidic extract.

		Control Brioche	Reduced-Fat Brioche	Control Rice Cake	Reduced-Fat Rice Cake
Weight loss (%)		8.43 ± 0.33 ^a^*	8.25 ± 0.98 ^a^	8.63 ± 0.20 ^a^*	9.18 ± 0.07 ^b^
pH					
	Dough	5.67 ± 0.06 ^a#^	5.62 ± 0.03 ^a^	7.00 ± 0.07 ^a#^	7.10 ± 0.15 ^a^
	Baked	5.52 ± 0.12 ^a^*	5.58 ± 0.07 ^a^	7.68 ± 0.05 ^a^*	7.67 ± 0.05 ^a^
a_w_		0.93 ± 0.00 ^a^*	0.94 ± 0.01 ^b^	0.94 ± 0.01 ^a^*	0.95 ± 0.01 ^b^
Texture					
	Hardness (N)	2.62 ± 0.38 ^a#^	2.89 ± 0.41 ^b^	2.20 ± 0.21 ^a^*	2.90 ± 0.25 ^b^
	Resilience (%)	10.85 ± 1.20 ^a^*	11.11 ± 1.42 ^a^	37.68 ± 2.46 ^a^*	33.49 ± 2.40 ^b^
	Cohesion (%)	34.97 ± 2.75 ^a#^	36.04 ± 2.90 ^a^	70.21 ± 2.76 ^a^*	66.31 ± 2.96 ^b^
	Springiness (%)	60.32 ± 4.74 ^a^*	60.92 ± 3.82 ^a^	85.77 ± 1.79 ^a#^	87.59 ± 1.33 ^b^
Color (Crumb)					
	L*	64.13 ± 2.95 ^a#^	64.30 ± 2.97 ^a^	60.73 ± 2.11 ^a#^	62.32 ± 1.66 ^b^
	a*	2.17 ± 0.39 ^a#^	2.70 ± 0.19 ^a^	1.09 ± 0.17 ^a#^	2.54 ± 0.18 ^b^
	b*	22.06 ± 1.84 ^a#^	20.37 ± 1.16 ^b^	26.67 ± 0.92 ^a#^	26.74 ± 1.00 ^a^
	∆L*	--	0.17 ± 2.97	--	1.59 ± 1.66
	∆a*	--	0.53 ± 0.19	--	1.45 ± 0.18
	∆b*	--	−1.69 ± 1.16	--	0.07 ± 1.00
	∆E	--	3.25 ± 1.66	--	2.75 ± 0.88
Moisture (%)		29.50 ± 0.31 ^a^*	28.44 ± 0.32 ^b^	39.68 ± 0.19 ^a^*	39.18 ± 0.43 ^b^
Ash (%)		1.03 ± 0.03 ^a^*	0.98 ± 0.01 ^b^	1.26 ± 0.02 ^a^*	1.28 ± 0.02 ^a^
Protein (g/100 g)		3.43 ± 0.11 ^a^*	3.42 ± 0.11 ^a^	3.44 ± 0.03 ^a^*	3.59 ± 0.10 ^a^
Total Fat (g/100 g)		11.31 ± 0.18 ^a#^	10.98 ± 0.11 ^b^	12.88 ± 0.23 ^a#^	11.41 ± 0.14 ^b^
Fatty Acids (% of fat)					
	SFA	46.5 ^a#^	45.3 ^b^	25.0 ^a^*	27.1 ^b^
	MUFA	39.0 ^a#^	40.3 ^b^	31.6 ^a^*	31.5 ^b^
	PUFA	14.5 ^a^*	14.4 ^b^	43.4 ^a^*	41.5 ^b^
Carbohydrates (g/100 g)		55.00 ± 0.48 ^a^*	56.18 ± 0.40 ^b^	42.71 ± 0.11 ^a^*	44.53 ± 0.43 ^b^
Energy (kcal/100 g)		335.54 ± 3.29 ^a*^	337.26 ± 1.28 ^a^	300.58 ± 1.41 ^a^*	295.16 ± 2.73 ^b^

Different letters in a row of the same product (brioche or rice cake) represent statistical differences (*t*-test, *p*-value < 0.05 (*), Mann–Whitney U, *p*-value < 0.05 (^#^)).

**Table 5:** Results obtained on the TPC, DPPH, and FRAP essays for the brioche (control and reduced-fat (RF) brioche) and rice cake (control and reduced-fat (RF) rice cake).

	Control Brioche	Reduced-Fat Brioche	Control Rice Cake	Reduced-Fat Rice Cake
TPC (mg GAE/g of extract)	1.55 ± 0.35 ^a#^	1.22 ± 0.27 ^b^	1.16 ± 0.33 ^a#^	1.29 ± 0.39 ^a^
DPPH inhibition (µmol AAE/g)	0.80 ± 0.45 ^a^*	0.95 ± 0.38 ^a^	1.02 ± 0.50 ^a#^	1.10 ± 0.61 ^a^
FRAP (µmol AAE/g)	2.04 ± 0.52 ^a#^	1.83 ± 0.27 ^b^	1.24 ± 0.35 ^a#^	1.39 ± 0.39 ^a^

Different letters in a row of the same product (brioche or rice cake) represent statistical differences (*t*-test, *p*-value < 0.05 (*), Mann–Whitney U, *p*-value < 0.05 (^#^)).

**Table 6:** Results of the triangle test for similarities between brioche and rice cake.

	Brioche	Rice Cake
Number of responses	34	36
Number of correct responses	14	19
Minimum number of correct answers (α = 0.05)	17	18

**Table 7:** Microbial analysis of the samples of reduced-fat brioche and rice cake and respective controls for the first four days after production.

Sample	Day	Total Viable Counts at 30 °C (cfu/g)	Molds at 25 °C (cfu/g)	Yeast at 25 °C (cfu/g)	*Bacillus* spp., (cfu/g)	*B. cereus*, (cfu/g)
Brioche control	1	<1.0 × 10^1^	<1.0 × 10^1^	<1.0 × 10^1^	<1.0 × 10^1^	<1.0 × 10^1^
	2	<1.0 × 10^1^	<1.0 × 10^1^	<1.0 × 10^1^	EN = 4.0 × 10^1^	<1.0 × 10^1^
	3	<1.0 × 10^1^	<1.0 × 10^1^	<1.0 × 10^1^	EN = 6.0 × 10^1^	<1.0 × 10^1^
	4	<1.0 × 10^1^	Present but <4.0 × 10^1^	<1.0 × 10^1^	<1.0 × 10^1^	<1.0 × 10^1^
Reduced-fat brioche	1	<1.0 × 10^1^	<1.0 × 10^1^	<1.0 × 10^1^	<1.0 × 10^1^	<1.0 × 10^1^
	2	<1.0 × 10^1^	<1.0 × 10^1^	<1.0 × 10^1^	<1.0 × 10^1^	<1.0 × 10^1^
	3	<1.0 × 10^1^	<1.0 × 10^1^	<1.0 × 10^1^	EN = 7.0 × 10^1^	Present but <4.0 × 10^1^
	4	EN = 9.0 × 10^1^	<1.0 × 10^1^	<1.0 × 10^1^	1.5 × 10^2^	<1.0 × 10^1^
Rice cake control	1	<1.0 × 10^1^	<1.0 × 10^1^	<1.0 × 10^1^	<1.0 × 10^1^	<1.0 × 10^1^
	2	Present but <4.0 × 10^1^	<1.0 × 10^1^	<1.0 × 10^1^	<1.0 × 10^1^	<1.0 × 10^1^
	3	<1.0 × 10^1^	<1.0 × 10^1^	<1.0 × 10^1^	<1.0 × 10^1^	<1.0 × 10^1^
	4	<1.0 × 10^1^	<1.0 × 10^1^	<1.0 × 10^1^	<1.0 × 10^1^	<1.0 × 10^1^
Reduced-fat rice cake	1	<1.0 × 10^1^	<1.0 × 10^1^	<1.0 × 10^1^	<1.0 × 10^1^	<1.0 × 10^1^
	2	<1.0 × 10^1^	<1.0 × 10^1^	<1.0 × 10^1^	<1.0 × 10^1^	<1.0 × 10^1^
	3	<1.0 × 10^1^	<1.0 × 10^1^	<1.0 × 10^1^	<1.0 × 10^1^	<1.0 × 10^1^
	4	<1.0 × 10^1^	<1.0 × 10^1^	<1.0 × 10^1^	<1.0 × 10^1^	<1.0 × 10^1^

Legend: EN: Estimated number.

## Data Availability

The original contributions presented in this study are included in the article. Further inquiries can be directed to the corresponding authors.
